# Cardiovascular Surgery Procedural Training and Evaluation: Current Status and Future Directions

**DOI:** 10.14797/mdcvj.1085

**Published:** 2022-06-03

**Authors:** Qasim Al Abri, Moritz C. Wyler von Ballmoos

**Affiliations:** 1 Houston Methodist DeBakey Heart & Vascular Center, Houston, Texas, US; 2 Weill Cornell Medicine, New York, US

**Keywords:** education, cardiac surgery, assessment, simulation based learning, transcatheter therapy, minimally-invasive surgery

## Abstract

The increasing complexity of heart disease manifestations and treatments as well as technological advancements make cardiovascular surgery an evolving specialty. In this review, we provide an overview of the factors leading to new developments in this field and discuss the adopted pathways to train cardiovascular surgeons in the United States. We also review the current challenges to the existing training culture and discuss the need to adopt adjuvant strategies to fulfill the societal expectations of what it means to be a competent cardiovascular surgeon.

## Introduction

Cardiovascular surgery is a young medical specialty. Open heart surgery as we know it today was unthinkable before the 1950s, when the first cardiopulmonary bypass circuits were devised.^1^ The technological advances and the expansion of surgical techniques that cardiovascular surgery has seen in this short time are truly remarkable. In parallel, major advances in diagnosis and medical management of cardiovascular pathologies, along with the emergence of interventional cardiology, have continuously moved the goal post for cardiac surgery. Prime examples are the development of percutaneous coronary interventions (PCI) in the 1980s and, more recently, the adoption of transcatheter technology for the treatment of valvular heart disease. With the establishment of interventional cardiology in the late 1970s, fewer residents pursued training in cardiovascular surgery for fear that the specialty would become obsolete. Yet cardiovascular surgery has not vanished. In fact, the country is facing an unprecedented national shortage of cardiothoracic surgeons.^2^

In many ways, cardiac surgery has been reinventing itself since its earliest days, and it must continue to do so as our population grows and ages while technological advances create new treatment paradigms. Furthermore, outcomes after cardiovascular surgery have significantly improved over time^3^ while accountability for outcomes has increased with public reporting.^4^ These changes have important implications for how we train cardiovascular surgeons today and adequately prepare them for future changes. This review discusses the current training pathways and requirements using the example of valvular heart disease, highlights recent developments in cardiovascular surgery that are creating new training needs, and suggests areas for improvement and further research.

## Training Pathways, Requirements, and Assessment Tools

The American Board of Thoracic Surgery (ABTS) has four pathways to board certification: completion of (1) a 5-year general surgery residency approved by the Accreditation Council for Graduate Medical Education (ACGME) followed by an ACGME-approved thoracic surgery residency; (2) a 5-year residency in general surgery, cardiac surgery, or vascular surgery accredited by the Royal College of Physicians and Surgeons of Canada and completion of an ACGME-approved thoracic surgery residency; (3) an ACGME-approved integrated 6-year program in thoracic surgery following medical school; or (4) an ACGME-approved 5-year vascular surgery residency followed by an ACGME-approved thoracic surgery residency. The specialty name and board designation of “thoracic surgery” as well as the names of related societies (Society of Thoracic Surgeons, American Association of Thoracic Surgery) allude to the common origin and shared areas of interest (organs of the chest) that encompass both general thoracic surgery and cardiovascular surgery. In fact, most ABTS-certified surgeons practice both general thoracic surgery and cardiovascular surgery. This prevailing practice pattern, the shared interests, and combined strength for health care policy purposes are important reasons why a separation of the two increasingly subspecialized disciplines has not yet occurred in the US. However, the benefits of dividing training and therefore board certification into two distinct disciplines, as is the case in Canada and most of Europe, is increasingly a topic of discussion.

**Figure 1:**
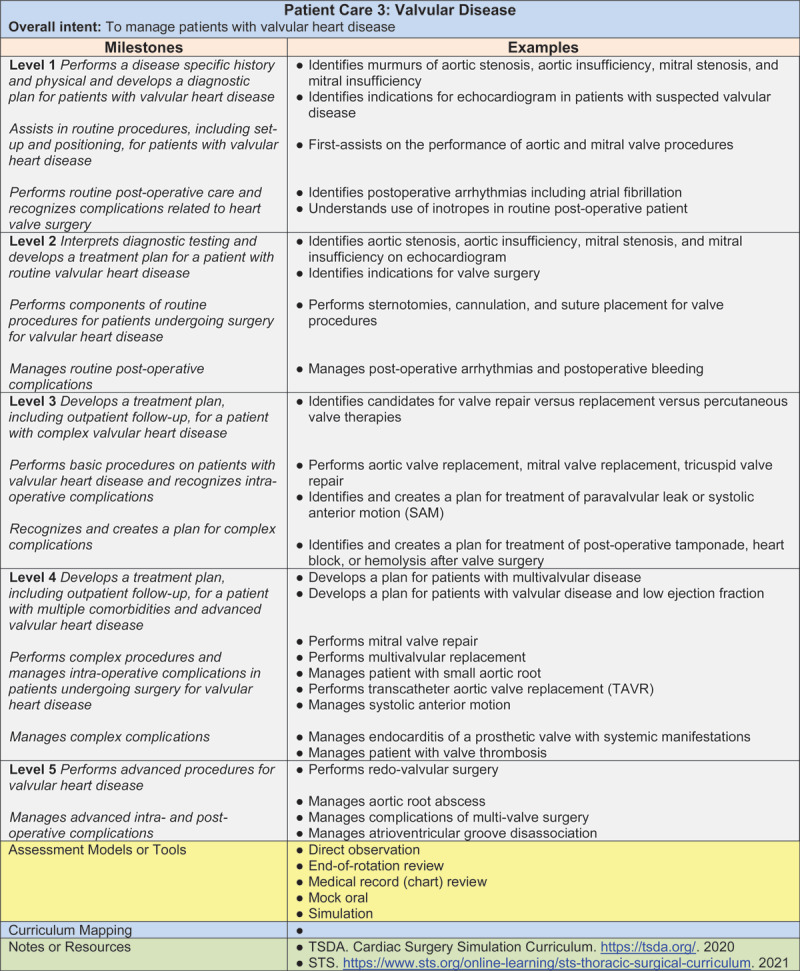
Accreditation Council for Graduate Medical Education milestone competencies for valvular disease. ©2020 Accreditation Council for Graduate Medical Education.

Currently, residents in the US are required to train and gain minimum proficiency in both general thoracic and cardiovascular procedures. The ABTS allows for a residency program focus with slightly different operative requirements weighted in either direction (general thoracic versus cardiovascular). The requirements are outlined in the ACGME Milestone Competencies, supplemented by the respective ACGME assessment forms. The ABTS also has a separate requirement for cases being performed when the resident is the surgeon.^5^ Both are rather rough guidelines to ensure a minimum level of competency. In the case of valvular heart disease, residents at graduation (level 4) are required to have a basic understanding of what valve pathology looks like on echocardiography, formulate appropriate treatment plans including surgical and transcatheter options (**Figure 3**), and be proficient in aortic/mitral/tricuspid valve repair and replacement surgery, multivalve surgery, complications of valve surgery, and transcatheter aortic valve replacement (TAVR) (**Figure 1**). The corresponding ABTS operative requirements are 25 aortic valve surgeries, 15 mitral valve surgeries, and 5 TAVR surgeries with another 10 as an assistant. The suggested assessment tools are direct observation, end-of-rotation review, medical record review, and simulation.

Considering recent developments in the structural heart disease domain, it is questionable if these requirements are sufficient to prepare future surgeons for practice. Similar concerns apply to all domains across general thoracic, cardiovascular, and congenital heart surgery. Considering the breadth of the field, competition for training time is unavoidable. In recognition of this, congenital heart surgery now has its own ACGME certificate that is obtained following a thoracic surgery residency, which currently has only a minimal requirement for congenital heart surgery.

## Challenges with Current Training Programs

It is noteworthy that the competencies set forth by the ACGME and ABTS are minimum requirements. Many residents graduate with higher operative numbers and more experience than outlined. On the other hand, a changing practice environment with increasing scrutiny of outcomes, public reporting, and reimbursement being tied to outcomes has changed both the training environment and realities for new surgeons starting to practice. Several studies support the notion that educational reform in cardiovascular surgery is very much needed, with many graduates being incompletely prepared to practice. In a study by Shah et al., residents were asked to report whether they routinely served as the operative surgeon for the most common cardiac surgical procedures.^6^ This study demonstrated profound heterogeneity in the operative experience of graduating residents. Graduating chief residents from all training pathways were surveyed, and nearly all reported that the only cardiac operations they routinely performed as the operative surgeon were coronary artery bypass grafting (CABG; 92%) and aortic valve replacement (AVR; 88%). In a different study, 16% of residents reported they had never performed an off-pump CABG procedure during residency, although 88% of them intended on doing so in practice,^7^ and up to 12% of residents reported logging operations inaccurately to meet minimum requirements for board certification.^8^

An increasing number of graduates also pursue additional training (roughly 50% in 2014, up from 10% in 2003).^9^ In a more current 2021 study by Bergquist et al., 40% of graduates reported additional training.^10^ This was closely tied to the length of their thoracic surgery residency, with those completing only a 2-year program most commonly pursuing additional training. Notably, few graduates from integrated 6-year thoracic surgery residency programs completed further training: 28% reported “inadequate training” as the reason for more training, 24% because it was required for a position, and 14% stated “personal reasons.”

## Heterogeneity in Cardiovascular Training

Further subspecialization of thoracic and cardiovascular surgery adds more complexity to the training issue. With significant developments over the last few decades and increasing overlap with other specialties, the idea of achieving competency in all domains is becoming more challenging. The vastly different training pathways, one being a 6-year program after medical school and the other a 2- or 3-year program following a surgical residency, add further heterogeneity in the way US residents are trained in cardiovascular surgery. In a survey that accompanies the annual in-training exam, 13% of residents reported no dedicated time in the cardiovascular surgery intensive care unit, and only 42% spent time in a cardiac care unit.^11^ While two-thirds of trainees had a cardiology service rotation, including interventional cardiology, one-third did not spend dedicated time with a cardiology team. The reported cardiovascular operative time increased in the senior years of training. However, dramatic differences exist, with a range of 0 to 52 weeks per year spent on a cardiovascular surgery rotation. Some of this variability is likely explained by different career pathways chosen (thoracic versus cardiac), but the difference between trainees across the board is nonetheless remarkable. Although this data is self-reported, similar heterogeneity in training and operative exposure has been described in other studies comparing the rotation schedule of US programs, again supporting the notion that significant variation exists in how residents are trained.^12^

## Keeping up with New Techniques and Technology

The advent of TAVR has completely changed the paradigm of how we treat aortic stenosis. Those suspicious of that notion are reminded that by 2017, the volume of TAVR procedures for the first time exceeded the combined volume of isolated surgical aortic valve replacement (SAVR) and SAVR plus coronary artery bypass grafting.^13^ This development is not confined to the aortic valve or to valvular heart disease alone. Distal aortic pathologies have been treated with endovascular solutions for a while, and new technology continues to move the target more proximally, now providing solutions for the ascending aorta and the aortic arch, which have previously been the undisputed domain of open surgery. Even cardiac and pulmonary support devices are increasingly being designed for minimally invasive percutaneous use. Cardiovascular surgeons have a unique opportunity to become the ultimate valve, aortic, or heart-failure specialist, with a deep understanding of diagnosis and treatment options (**Figure 3**) and the ability to perform the full spectrum of procedures and manage complications thereof. Nonetheless, the question still remains: Can the necessary procedural training and such disease-specific competency truly be obtained during a thoracic surgery residency? Unlikely, unless it is at the cost of training in other areas.

In the case of structural heart disease and transcatheter valve therapies, professional societies offer no guidance or consensus in general on the requirements and competencies that must be met, apart from very basic regulatory mandates.^14^ Procedural training and assessment in this arena currently are being offered in various forms, including nonaccredited postgraduate training programs; Food and Drug Administration-mandated industry-sponsored device-specific training courses using case discussions and simulation; industry-sponsored physician proctorship; and society-sponsored national conferences with simulation and case discussion.

**Figure 2:**
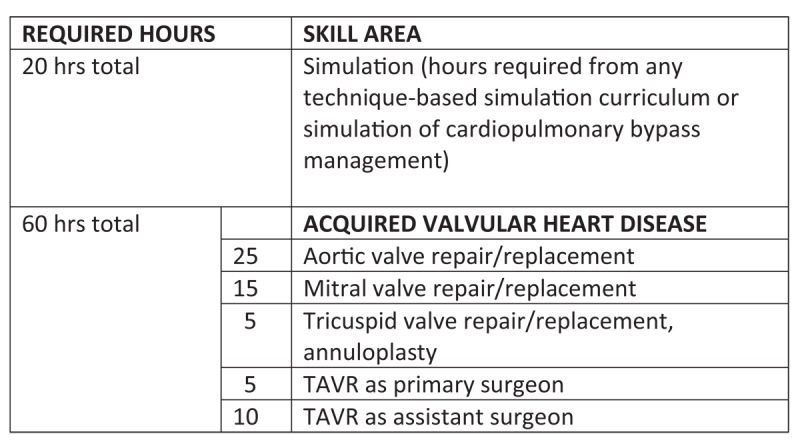
American Board of Thoracic Surgery requirements for simulation & acquired heart valve disease. TAVR: transcatheter aortic valve replacement.

In recent years, TAVR training and wire skills have also been incorporated into thoracic residency; however, the required exposure and case experience are minimal (**Figures 1, 2**). For example, the ABTS requires residents in the cardiovascular track to complete 5 TAVR cases as the primary operator (an additional 10 as an assistant); 15 interventional wire-based procedures, including 5 left heart catheterizations, percutaneous coronary interventions, thoracic endovascular aortic repairs (TEVAR), or transcatheter edge-to-edge repair; and 10 intra-aortic balloon pump placements.

**Figure 3:**
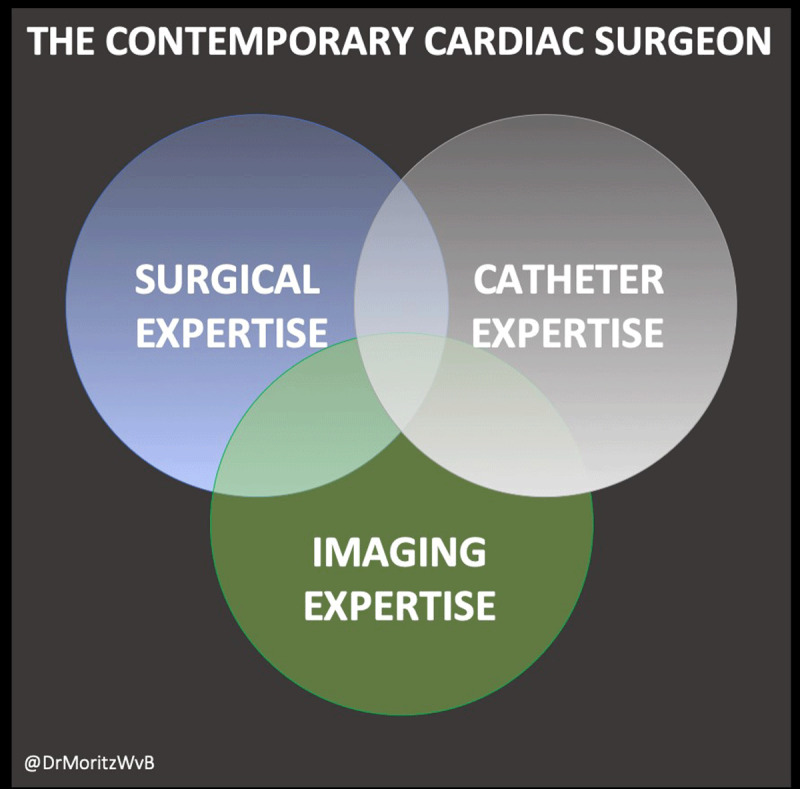
Triad of required expertise to master the full spectrum of therapeutic options in cardiovascular surgery.

Residency programs that have embraced the rapidly occurring changes in structural heart disease treatment and management likely will graduate cardiovascular surgery trainees with sufficient experience to perform TAVR in conjunction with their cardiology partners as an equal member of the structural heart team. On the other hand, the already existing heterogeneity and gaps in training may widen further with the introduction of new technology and techniques across the spectrum of thoracic and cardiovascular surgery. As it stands now, only a minority of residents training in cardiovascular surgery routinely perform transcatheter therapies as part of their training (TAVR 36%; TEVAR 29%).^6,15^ Off-pump CABG, minimally invasive valve operations, and transcatheter aortic interventions were rarely performed by graduating residents as the operative surgeon (TAVR 36%; TEVAR 29%; MVr 40%; miniAVR 33%; and miniMVrR 13%).^6^

The same applies to new techniques in cardiovascular surgery. Much of the new technology in the cardiovascular space aims at reducing the invasiveness of interventions. As such, the success of transcatheter therapies has caused a renewed interest and push for less-invasive surgical approaches, similar to what has happened in other surgical disciplines. Adoption of minimally invasive cardiac surgery (MICS) overall has doubled over the last decade, although implementation of these techniques has been much higher in Europe than in the US. There, roughly 50% of isolated mitral valve surgery is being performed using MICS techniques. By comparison, in the US it is about 20%.^16^ An often-posed question is why US adoption of MICS has not been higher. The transatlantic differences may be explained in part by differences in reimbursement models and the medicolegal environment. Comparisons are also made to general surgery and thoracic surgery, where the standard of care is often an endoscopic minimally invasive approach. In our opinion, that comparison is not entirely fair as other surgical disciplines do not require anticoagulation, the use of a heart-lung machine, and cardiac ischemic time. Yet, MICS can be taught and learned safely and becomes cost-neutral and beneficial compared to standard sternotomy techniques after an initial learning curve.^17,18^ Only a small minority of trainees in the US currently have the opportunity to perform MICS in the role of surgeon (MICS AVR 33%; MICS mitral valve surgery 13%).^6^

Whether transcatheter therapies or MICS, the biggest challenge arguably remains the additional learning curves that must be mastered. Moving from more to less invasive also typically means trading tools and techniques of visualization. To be successful, one must have not only good dexterity but also competency and experience with multimodality imaging in the preprocedural and procedural phases of care. In other words, expertise is required in traditional surgical skills, imaging, and catheter-based skills to fully implement these new techniques.

## Areas for Improvement in Cardiovascular Surgery Education

We suggest that future efforts focus on four areas to improve cardiovascular surgery education: (1) education research (standardization and validation of assessments); (2) simulation (procedural skills); (3) surgical coaching (ongoing refinement of skills and education beyond residency); and (4) subspecialty certification (disease-specific fellowships).

Although dated, the traditional apprenticeship model of surgical education is still widely prevalent. The ACGME suggests using direct observation, end-of-rotation review, and medical record review to assess residents. Evaluation of surgical skills by master surgeons is key for providing trainees with feedback. However, the process for how this feedback is provided is left open to interpretation by, or personal preference of, the individual surgeon, and it is often devoid of clear metrics or standards.

### Assessments

A review of 292 published articles on surgical education found that the majority were editorial in nature, and less than 5% had experimental data to support claims.^19^ Beyond the lack of appropriate research questions, many papers have grave methodological issues, such as missing an appropriate control group (56%) or an actual results section altogether (54%). In a recent study, Luckoski et al. set out to create an inventory of assessment tools in use across several US surgical residency programs.^20^ They identified 42 unique assessment tools, of which only about 10% were used by more than just one program. Of all assessments, 60% were used monthly or less frequently. Two-thirds of instruments were retrospective global assessments rather than discrete observed performances. Only 10% of the assessment tools had established reliability or validity evidence. In other words, programs predominantly employ nonstandardized global assessment tools that lack reliability or validity evidence. This calls into question the usefulness of current tools to assess performance and progress, which make it nearly impossible to establish competency standards across programs. There are metrics that could be used for a more reliable and standardized assessment. Examples are motion trackers to measure operator efficiency, pressure sensors tracking the force applied to tissue and instruments, or instrument kinematic tracking data for robotic platforms. Another example is radiofrequency tagging of surgical instruments that can be leveraged to measure frequency of instrument exchanges or efficiency for specific portions of a procedure. Based on this evidence, medical education programs need better tools to track training progress and identify gaps as well as tailored solutions for closing them. They also need to identify and validate objective simulation-based metrics to assess and monitor the progress of trainees throughout their training.

### Simulation

A large body of evidence supports the use of simulation in surgical education. For example, substantial improvements in coronary anastomosis performance can be observed following simulation and deliberate practice.^21^ Blinded assessment by faculty at the beginning, midpoint, and end of the simulation using video recording of the procedure can document the progress and provides a structured, low-stake environment for skills assessment and feedback for trainees. This has proven to be an extremely effective tool, regardless of the fidelity of the simulator. Computer and material science technology will continue to develop new tools that can be leveraged for educational purposes.

A randomized clinical trial and subsequent cohort study have demonstrated the effectiveness of hands-on and virtual reality simulation to train residents in endovascular procedures; residents were shown to have improved competency based on objective metrics, and the training reduced the chances of the attending taking over when the resident applied these skills in the actual clinical setting.^22,23^ ABTS now has a 20-hour simulation requirement that applies to all residents, and the Thoracic Surgery Directors Association provides resources and links to simulation training, including commercially available surgical simulators. How that time is used is entirely up to the training programs and likely varies greatly. This variation highlights the need for a structured and validated simulation curriculum that can be implemented at reasonable cost at all training institutions. Furthermore, the annual in-training examination should consider adding a skills assessment in addition to assessing overall knowledge.

The traditional concept is that residency prepares its trainees for safe and independent practice, and that graduation occurs when those requirements are met. In reality, this assumption is becoming increasingly more difficult for all the reasons stated above. Surprisingly, coaching has played almost no role in surgery other than an informal one. Professional athletes, musicians, and executives rely heavily on coaches. No professional marathoner shows up to a race without their coach, and it is not because they don’t know how to run. Why, then, would surgeons be exempt from that opportunity to achieve peak performance, continued growth, and pivoting when needed.

### Coaching

Several studies have explored video-based coaching in surgery. The process typically matches an individual surgeon in practice with a surgical colleague who has been trained in the core principles of coaching. As part of the coaching, feedback on technical skills, cognitive skills, and decision making is provided. This concept is still novel in surgery and occasionally is met with skepticism, but it is gaining acceptance as a method of surgical education. As more surgeons look toward video-based coaching for quality improvement, a consistent definition of these programs, goals, and metrics for assessment will need to be established.^24^ In addition to its value for recent graduates, surgical coaching also could provide an important support system for surgeons returning to work after an absence or for those starting new procedures. Overall, surgical coaching could become an important adjunct to support graduates and early career faculty with performance improvement opportunities, although the required investment in infrastructure and coaches is a potential hurdle.

### Fellowships and Certification

Finally, disease-specific fellowships and certification following thoracic residency could provide a structured avenue to gain comprehensive procedural skills and knowledge. Such training opportunities exist today, and various institutions offer fellowships in structural heart disease, transcatheter therapies, aortic disease, or advanced heart-and lung support and transplantation. However, there is currently no oversight or defined structure for such nonaccredited programs, and a great deal of variation exists in terms of duration, responsibilities, and expectations between institutions.^14^ As such, these fellowships provide a temporary solution to afford graduates an opportunity to refine their skill set or acquire deeper expertise in a field, but they likely will be insufficient to keep up with the rapid evolution of treatment paradigms in cardiac surgery. Still, domain-specific fellowships and certification may provide the most realistic platform to acquire advanced skills beyond procedures that are routinely performed by cardiovascular surgeons.

## Conclusion

Cardiovascular surgery is a very dynamic and evolving specialty due to the rapid innovations and advancements in this field. Cardiovascular surgical training programs must adapt with these changes and adopt the necessary steps to graduate qualified cardiovascular surgeons with various skill sets who are able to successfully use all available options to provide the best care for patients.

## Key Points

- The field of cardiovascular surgery is rapidly evolving despite it being a relatively new specialty, and cardiovascular training programs should consider including advanced technologies and therapies to their curricula.
- There is a great need to adopt simulation in the training and assessment of cardiovascular residents.
- A modern-day cardiovascular surgeon should be familiar with newer therapies including minimally invasive approaches and transcatheter procedures.
- Future efforts to improve cardiovascular surgery education should focus on standardizing and validating assessments, adding simulation of procedural skills, surgical coaching with ongoing refinement of skills and education beyond residency, and subspecialty certification such as disease-specific fellowships.
